# The Action of Certain Somnifacients on the Horse1Read before the Pennsylvania State Veterinary Medical Association, March 7, 1900.

**Published:** 1900-05

**Authors:** E. Stanton Muir

**Affiliations:** Philadelphia, Pa.


### THE ACTION OF CERTAIN SOMNIFACIENTS ON THE HORSE.[1]

By E. Stanton Muir, Ph.G., V.M.D.,

PHILADELPHIA, PA.

(Concluded from page 198.)

The line of experiments with the next drug, Indian Cannabis, was extremely interesting, as there was a difference in its action on different animals. The preparation used was P. D. & Co.'s N. L., which is an assayed fluid extract and a very reliable preparation.

Experiment No. 8. The first animal used was a bay gelding, eight years old, fifteen and three-quarters hands high. Temperature 100.8° F., pulse 42, respirations 16; heart strong and regular; general condition was poor. I gave him an intravenous in-

[1] Read before the Pennsylvania State Veterinary Medical Association, March 7, 1900.

jection of 12.5 c.c. of the normal liquid, undiluted, at 12.20 P.M. In a few moments he became very nervous. At 12.42 the pulse was almost imperceptible, respirations normal, and heart 36. At this time he received a second 12.5 c.c., when he became hypersensitive. If he was touched, however lightly, with a straw, he would become excited and resent it. At 12.50 sleepiness was marked, and the stupor continued without any other symptoms until 1.15, when I again injected 15 c.c. into the veins. At 1.25 the temperature dropped 0.2 of a degree, and he was sweating profusely over the whole body. At 2 he was standing, twitching, and jerking the head as if dreaming, was still sweating, and his temperature had dropped to 99° F. At 2.20 he began to awaken, and at 3.21 hyperæsthesia was pronounced, but when not annoyed was very sleepy, and his temperature had dropped to 96° F., the lowest point it reached. At 4.30 P.M. his temperature had risen to 97° F., and he was decidedly delirious, in which condition he continued until 10 P.M., when he was lying down eating hay, apparently none the worse for his experience. A fact noticed was: as the sweating increased the temperature fell, and when it ceased the heat of the body slowly rose to normal.

The *prominent symptoms* were pronounced sleepiness, sweating, fall of temperature, delirium, and hyperæsthesia.

EXPERIMENT No. 9. A small horse, almost a pony, fourteen hands high, weighing 575 pounds. Temperature, 98° F.; pulse, full and strong; respirations 12; heart 42 and regular; condition fair. At 11.35 A.M. gave him an intravenous injection of 15 c.c. of N. L. Indian Cannabis, undiluted. In two minutes he stopped eating and became slightly delirious. At 11.45 the muscular system began to relax, the penis was protruded, and he was standing braced against the side of the stall, sleeping soundly. At 12.10 he fell down, and continued to sleep soundly until 6 o'clock the next morning, a period of eighteen hours, with the exception that at 2 P.M. he arose and immediately lay down and went into a sleep from which it was impossible to awaken him. There was not the slightest sweating, but his temperature went down to 92.5° F. up to 4.50 P.M., after which it slowly rose, and at 11.50 P.M. was 97.4° F. The pulse became very slow and shallow, respirations became irregular, and the heart dropped to 30 during the experiment.

The only *prominent symptoms* were sleep and the drop in temperature. The next morning he ate his feed and hay, apparently benefited by his sleep.

To those of you who have read Dr. H. C. Wood's description of his personal experience with Indian Cannabis, will, after you have heard the result of the next experiments, conclude as I did, that the action of this drug is the same on the horse as it is on man. The action in these three experiments was that of a deliriafacient, while the narcotic action was *nil* or very slight.

Experiment No. 10. Gray gelding, eight years old, fifteen and one-half hands high, weighing 800 pounds. Pulse full and normal, respiration 7, heart 52 and fairly strong; temperature normal; general condition poor. At 2.55 gave an intravenous injection of 30 c.c. of the N. L. Indian Cannabis, and in five minutes he was swaying from side to side. At 3.15 he was standing quiet, but would not allow anyone to take his temperature, becoming very violent, trying to kick, bite, or strike when approached, and was snorting constantly up to 4.30, after which the symptoms gradually abated. He remained irritable, however, until the next morning.

The *prominent symptoms* were a slight sleepiness and muscular weakness at first, and shortly becoming very irritable, but no diaphoresis.

Experiment No. 11 showed a still more violent action from the same amount of the drug administered in the same manner to a larger horse and one that was in good condition. Gray gelding, twelve years old, sixteen and a quarter hands high, weighing over 1000 pounds. Temperature 99.8° F., pulse full and regular, respirations 10, heart 38, regular and strong. At 11.35 A.M. 30 c.c. were thrown into the vein, and in six minutes muscular twitchings started, and he became extremely hypersensitive. At 11.50 he was sweating profusely, and was snorting and too irritable to approach. At 1 the muscular symptoms had abated somewhat; he was constantly moving from one side of the stall to the other, and was irritable and excited. At this time the respirations became labored and even stertorous; the mucous membranes were anæmic, but it was impossible to make close examinations. At 2 P.M. he was still very delirious, and the sweat was drying up. At 4 P.M. a secondary sweating started, but did not last long. He continued to be irritable and slightly delirious until 11.50 P.M., when his condition became more normal. The next morning there were no symptoms to show that he had undergone such an experience.

The *prominent symptoms* were hyperæsthesia, sweating, irritability.

All the former experiments being performed on horses that were standing tied in open stalls I decided to try the next on a horse loose in a box-stall. The stall was 12 x 12 feet.

EXPERIMENT No. 12. Gray gelding, sixteen and a half hands high, weighing 1050 pounds and in very good condition. Temperature 100.8° F., pulse full and regular, respirations normal, and heart regular and strong. I injected 45 c.c. (f℥jss) of the normal liquid into the jugular at 11.22 A.M, and had an experience that is vivid to me to this day. He became restless in two minutes, and in three minutes was rearing, kicking, and snorting like a mad horse. At 11.30 he was sweating profusely and running along the wall always on one side of the stall, same as does a lion in a cage before feeding-time. At 11.45 the steam was coming from him in clouds, and the sweat was running from his belly in a stream. He was too violent to approach—in fact, we dare not open the door of the stall, or he would come at us in a manner not pleasant. At 12 noon he seemed oblivious to pain, as his face was badly cut and bleeding by contact with the wall, still he continued running back and forward as before. There was some cob-corn in the feed-box, and he would take a mouthful and not appear to know that he was chewing it. At 2.50 P.M. he was injuring himself so badly that he was lassoed and tied to the sides of the stall. He would strike and bite at anyone who approached, and kick with both hind feet when attempts were made to take his temperature. I should like to have taken his temperature, as I am sure it would have been much below normal. From this time until 4.30 he continued in about the same condition, standing quiet, but becoming very violent when approached or annoyed. The sweat began to dry up at 4 P.M., but he continued to be more or less delirious and excitable, not eating anything until the next morning, and even all next day he was more nervous and excitable than he normally was.

The *prominent symptoms* were delirium, sweating, excitable condition.

EXPERIMENT No. 13. While the last experiment was going on I had a small horse in the next box-stall under the influence of the same drug, which showed a line of symptoms exactly opposite from those we have just read. This was on a small bay gelding, fourteen and a half hands high, weighing 650 pounds. Temperature 100° F., pulse 36 and weak, respirations normal, heart regular but not strong. At 11.33 I injected 30 c.c. ; he became staggery in four minutes, and at 11.45 was standing sleeping, and the muscular system had begun to relax. At 11.47 he fell down on his knees, but recovered, and stood up until 12.03, when he fell down and went into a dead sleep. His temperature had now fallen to 99.4° F., but there was no sweating. At 12.30 he was still sleeping soundly, and the temperature was down to 97.4° F. At 1.30 he was sleeping soundly, no delirium, heart 56 and strong, respirations 12 and deep, pulse full, and temperature had fallen to 95° F. The lowest point recorded by the thermometer was 91.8° F. at 4.30, when he was still dead asleep. At 5 the temperature had risen to 93.5° F., and it continued to rise gradually until 6 the next morning, when he awoke, seeming all right except a slight muscular weakness.

The *prominent symptoms* were sleep, muscular relaxation, and fall in temperature.

EXPERIMENTS No. 14 and 15 were on a gray gelding and a bay gelding, the first being rendered exceedingly vicious by receiving 15 c.c. intravenously, so bad in fact that it was impossible to take his temperature; he did not sweat, nor did he show any signs of sleepiness. The bay gelding, about the same size and in the same condition as the former, was put sound asleep in twenty-three minutes' time with 25 c.c., from which he did not awaken for 3.25 hours, and then not the least excitable. After the acute symptoms here reported had passed off both animals gradually assumed their normal condition, apparently none the worse from their experience.

The *prominent symptoms* shown in all the experiments with Indian Cannabis were loss of temperature and hyperæsthesia. While in some we note extreme delirium and in others sleep pure and simple, some sweating profusely and others showing no diaphoretic action, sweating seems to have had nothing to do with the fall of temperature, for we note a fall in each case, and the greatest drop in the case which did not sweat at all. Action on the heart *nil*, or if any it has a stimulant action.

In making a summary of the whole line of experiments, we find that sulphate of morphia always produces more or less delirium in the horse, much more than that noticed on man, while we occasionally note delirium following the administration of large doses of Indian Cannabis. Pupils are widely dilated after large doses of morphia. Indian Cannabis has no action in this direction, chloral has no action on the pupil, and the only pronounced action noticed was somnolence, the apparent muscular weakness being no doubt secondary to the hypnotic action of the drug. In the case of morphia sulphate it will be noted that the mydriatic action came on only after large quantities of the drug had been administered and shortly after the nervous symptoms appeared. The toxic dose of fluid extract of Indian Cannabis must be very large, as 60 c.c. (f℥j$\frac{1}{3}$)

given intravenously did not produce any alarming symptoms; small doses seem to stimulate some part of the sensory nervous system, while large doses paralyze. The similarity between the symptoms shown by Indian Cannabis in these experiments and those recorded by Dr. H. C. Wood in the description of his personal experience with the drug is noticeable: the muscular weakness, the seeming exaggeration of distances, sound sleep, local anæsthesia, and no bad after results. There is one other point on which I wish to speak and that is in reference to the largest amount of each of these drugs that can be given with safety. Of course, the size and condition of the horse have a great influence in this direction, but I infer from the experience here obtained that on an average-sized horse, say of 1100 pounds weight, in good condition, it would be safe to give of sulphate of morphia, 1 gramme (15½ grains), of fluid extract of Indian Cannabis, 50 c.c. (f℥j and fʒv), and of chloral 25 to 30 grammes (ʒvj to ℥j), dissolved in distilled water and injected slowly into the jugular. Do not understand me to mean that these are the proper therapeutic doses, but the limit so far as safety goes.

